# Discovery of a New Human Polyomavirus Associated with *Trichodysplasia Spinulosa* in an Immunocompromized Patient

**DOI:** 10.1371/journal.ppat.1001024

**Published:** 2010-07-29

**Authors:** Els van der Meijden, René W. A. Janssens, Chris Lauber, Jan Nico Bouwes Bavinck, Alexander E. Gorbalenya, Mariet C. W. Feltkamp

**Affiliations:** 1 Department of Medical Microbiology, Leiden University Medical Center, Leiden, The Netherlands; 2 Department of Dermatology, Jeroen Bosch Hospital, ‘s-Hertogenbosch, The Netherlands; 3 Department of Dermatology, Leiden University Medical Center, Leiden, The Netherlands; University of Michigan, United States of America

## Abstract

The *Polyomaviridae* constitute a family of small DNA viruses infecting a variety of hosts. In humans, polyomaviruses can cause infections of the central nervous system, urinary tract, skin, and possibly the respiratory tract. Here we report the identification of a new human polyomavirus in plucked facial spines of a heart transplant patient with trichodysplasia spinulosa, a rare skin disease exclusively seen in immunocompromized patients. The trichodysplasia spinulosa-associated polyomavirus (TSV) genome was amplified through rolling-circle amplification and consists of a 5232-nucleotide circular DNA organized similarly to known polyomaviruses. Two putative “early” (small and large T antigen) and three putative “late” (VP1, VP2, VP3) genes were identified. The TSV large T antigen contains several domains (e.g. J-domain) and motifs (e.g. HPDKGG, pRb family-binding, zinc finger) described for other polyomaviruses and potentially involved in cellular transformation. Phylogenetic analysis revealed a close relationship of TSV with the Bornean orangutan polyomavirus and, more distantly, the Merkel cell polyomavirus that is found integrated in Merkel cell carcinomas of the skin. The presence of TSV in the affected patient's skin was confirmed by newly designed quantitative TSV-specific PCR, indicative of a viral load of 10^5^ copies per cell. After topical cidofovir treatment, the lesions largely resolved coinciding with a reduction in TSV load. PCR screening demonstrated a 4% prevalence of TSV in an unrelated group of immunosuppressed transplant recipients without apparent disease. In conclusion, a new human polyomavirus was discovered and identified as the possible cause of trichodysplasia spinulosa in immunocompromized patients. The presence of TSV also in clinically unaffected individuals suggests frequent virus transmission causing subclinical, probably latent infections. Further studies have to reveal the impact of TSV infection in relation to other populations and diseases.

## Author Summary

Diseases that occur exclusively in immunocompromized patients are often of an infectious nature. Trichodysplasia spinulosa (TS) is such a disease characterized by development of papules, spines and alopecia in the face. Fortunately this disease is rare, because facial features can change dramatically, as in the case of an adolescent TS patient who was on immunosuppressive drugs because of heart-transplantation. A viral cause of TS was suspected already for some time because virus particles had been seen in TS lesions. In pursuit of this unknown virus, we isolated DNA from collected TS spines and could detect a unique small circular DNA suggestive of a polyomavirus genome. Additional experiments confirmed the presence in these samples of a new polyomavirus that we tentatively called TS-associated polyomavirus (TSPyV or TSV). TSV shares several properties with other polyomaviruses, such as genome organization and proteome composition, association with disease in immunosuppressed patients and occurence in individuals without overt disease. The latter indicates that TSV circulates in the human population. Future studies have to show how this newly identified polyomavirus spreads, how it causes disease and if it is related to other (skin) conditions as well.

## Introduction

Members of the polyomavirus family (*Polyomaviridae*) infect mammals (rodents, bovines, primates) and birds (fowl, psittacines), and can affect various organs. So far five human polyomaviruses have been described. Two of these, JC-polyomavirus (JCPyV or JCV) and BK-polyomavirus (BKPyV or BKV), are established pathogens in immunocompromized hosts causing progressive multifocal leukoencephalopathy in AIDS patients and nephropathy in renal transplant recipients, respectively. In 2007, two additional human polyomaviruses were described, KI-polyomavirus (KIPyV or KIV) and WU-polyomavirus (WUPyV or WUV) [1], [2], which were isolated from the respiratory tract and whose pathogenicity is still unclear. The most recently discovered human species concerns the Merkel cell polyomavirus (MCPyV or MCV) found to be integrated in a large proportion of Merkel cell carcinomas of the skin [3], but detected in apparently healthy skin, plucked eyebrow hairs and other cutaneous carcinomas as well [4]. The transforming, oncogenic potential of polyomaviruses was recognized long ago in rodents following natural infection, and after experimental infections with JCV or BKV causing tumors in newborn hamsters [5], [6]. Here we describe the identification of a new human polyomavirus that combines specific properties of other human polyomaviruses, as it infects the skin and seems to cause disease only in immunocompromized patients probably as the result of unrestricted virus and host cell proliferation, possibly the inner root sheath cells of hair follicles.

Trichodysplasia spinulosa (TS), also known as pilomatrix dysplasia, cyclosporine-induced folliculodystrofy or virus-associated trichodysplasia, is a rare skin disease characterized by the development of follicular papules and keratin spines known as spicules [7], [8], [9], [10], [11], [12], [13], [14]. The lesions are most striking in the face, especially on the nose, eyebrows and auricles, but other parts of the body can be affected as well. The disease is accompanied by thickening of the skin and alopecia of eyebrows, sometimes also of lashes and scalp hairs, in some cases leading to distortion of facial features and a leonine appearance [8]. Histologically, TS is characterized by distended and abnormally maturated hair follicles with high numbers of inner root sheath cells containing excessive amounts of trichohyalin [7], [8], [9], [10], [11], [12], [13], [14]. From these aberrant follicles the keratin spicules originate that become 1–3 mm in length.

TS is exclusively found in immunocompromized patients, such as solid organ transplant recipients and acute lymphocytic leukemia patients [7], [8], [9], [10], [11], [12], [13], [14]. Initially, the condition was described as a side-effect of cyclosporine treatment, but later it was also observed in patients treated with other immunosuppressive or chemotherapeutic drugs. In analogy with other diseases exclusively occurring in immunocompromized patients, an infectious etiology was suspected. In 1999, Haycox and coworkers for the first time by transmission electron microscopy (TEM) demonstrated the intracellular presence of virus particles probably belonging to the papovavirus family [8]. Since 2000 the papovaviruses are classified in separate families, the *Papillomaviridae* and *Polyomaviridae*. In subsequent TS case reports, the presence of crystalloid arranged clusters of 40-nm virus particles preferentially in the nuclei of inner root sheath cells was confirmed [11], [13], [14]. Shape, size and localization suggested the presence of a small, non-enveloped DNA virus, likely a polyomavirus, but attempts to culture or detect the virus using PCR methods based on polyomavirus or papillomavirus-specific primer sets failed [8], [13], [14].

Here we describe a new case of TS and report the amplification, cloning and identification of a new human polyomavirus isolated from TS spicules. This virus was provisionally called TS-associated polyomavirus (TSPyV or TSV) and phylogenetic analysis was performed to determine its evolutionary position among the other known polyomaviruses. To investigate the putative causal relationship between TSV infection and TS, the clinical response after treatment with the anti-viral drug cidofovir was monitored, and TSV-specific quantitative PCR was developed to measure the TSV load in clinical samples before and after antiviral treatment. With this new PCR we also estimated the TSV prevalence in a group of unaffected immunosuppressed patients and provide evidence for TSV circulation outside TS patients as well.

## Results

### Case description

**Figure 1:**
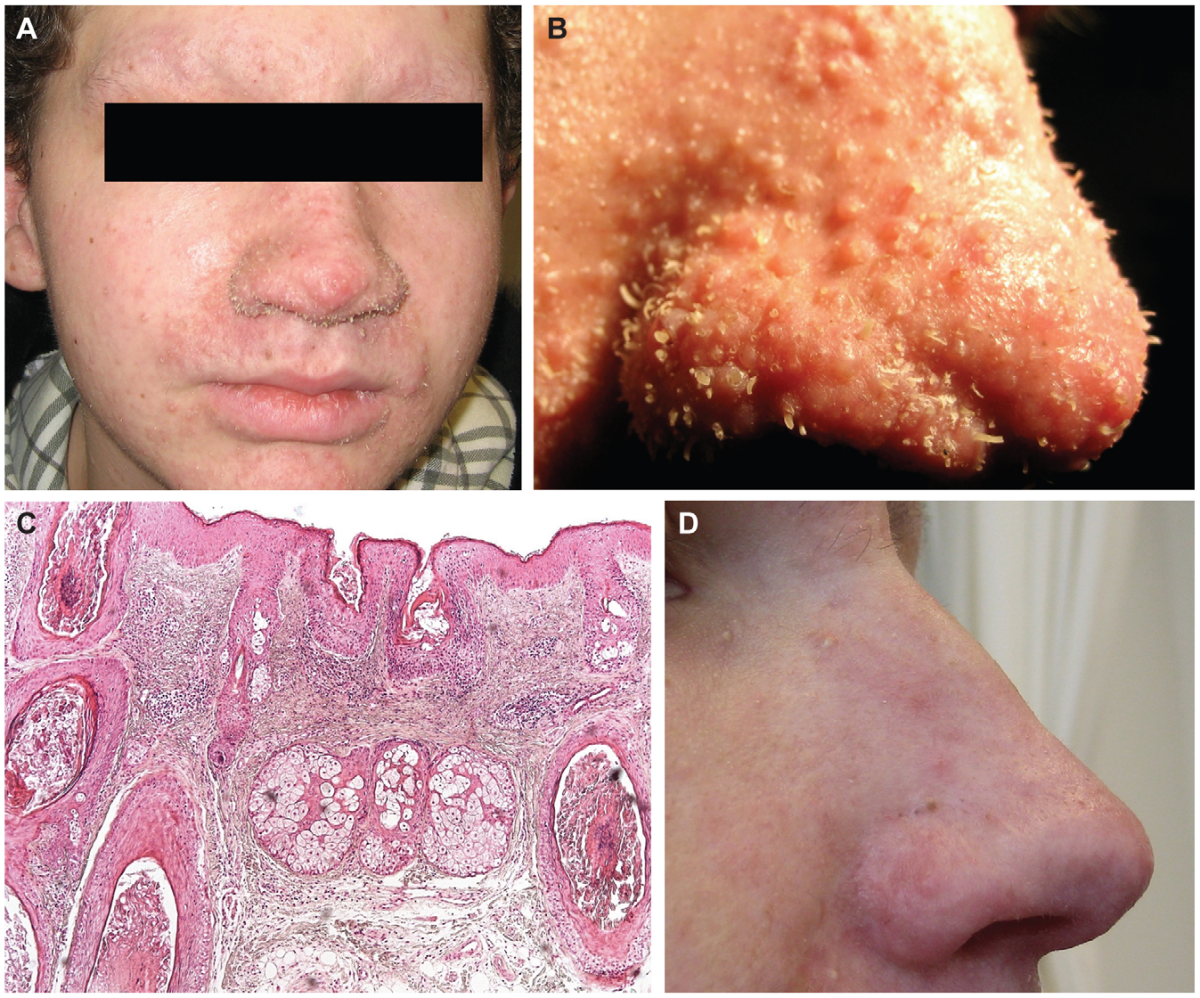
Clinical appearance and histology of the trichodysplasia spinulosa patient. Facial appearance at presentation is shown in panel **A**. Note the thickened skin, particularly on the nose and in the eyebrow region accompanied by central alopecia. Apart from the eyebrows and nose, papules are seen on the cheeks, chin, forehead and ears. Especially on the nose, but occasionally also in cheeks and chin, keratotic spicules protruded from the enlarged follicular orifices. Panel **B** shows a close-up of the nose at presentation with numerous papules and spicules. In panel **C** is shown a section of a formalin-fixed, paraffin-embedded biopsy of a hyperkeratotic follicular papule from the forehead. The epidermis reveals enlarged, hyperplastic hair bulbs and hypercornification within a distended follicular infundibulum (HE stain, 10×). Panel **D** shows a detail of the nose 3 months after topical cidofovir treatment. Papules and spicules have largely resolved and hairs have regained growth.

In the late spring of 2009, a 15 years old Caucasian male heart transplant patient was seen in the Dermatology outpatient clinic of the Jeroen Bosch Hospital because of spots and spines in the face. One and a half year prior to presentation, a year after transplantation and start of immune suppressive treatment, his skin condition had started to develop with desquamation of the eyebrows, gradually followed by the development of follicular, skin-colored, indurated papules on the eyebrows, nose, ears, malar-region and forehead (**Figure 1A**). Subsequent symptoms were loss of eyebrow hairs and partially of the eyelashes. From the enlarged follicular orifices, small hyperkeratotic white-yellowish spicules started to protrude on the eyebrows, nose and ears (**Figure 1B**). Comparable solitary hyperkeratotic papules and spicules also developed on the legs. Over a period of one year, as skin of his ears, eyebrows and nose had thickened, his overall facial appearance had changed dramatically.

In the end of 2006 he had been transplanted elsewhere for dilated cardiomyopathy of unknown cause and was placed on immunosuppressive treatment. Transplantation was complicated by a cerebrovascular event and epilepsy due to embolization from the left ventricle. A year after transplantation he was treated for an EBV-positive large B-cell lymphoma with rituximab and lowering of the immunosuppressive treatment. At presentation in 2009, apart from the immunosuppressive regimen (tacrolimus 2.0 and 1.5 mg daily; mycophenolate mofetil 750 mg 2 dd; methylprednisolone 10 mg 1 dd), he was also using amplodipine (calcium-antagonist), pravastatine (statin) and levetiracetam (anti-epilepticum).

As the patient refused the taking of biopsies, an hematoxylin-eosin (HE)-stained section was retrieved, prepared from a biopsy taken previously of a hyperkeratotic papule from the eyebrow region, as well as a snap-frozen fragment thereof. The HE section showed substantially distended and enlarged hair follicles (**Figure 1C**). Some hair bulbs were hyperplastic and bulbous, encroaching on hair papillae that were diminished in size. Some hair follicles showed presence of poorly formed hairs. Attempts to demonstrate the presence of TSV particles by TEM in the biopsy fragment failed because of poor sample quality.

The constellation of findings was diagnostic of viral-associated TS in an immunosuppressed patient. Based on this diagnosis and the assumption that a polyomavirus was causing the disease, the patient was started on topical cidofovir 1% cream treatment twice a day. Gradually over the first three months the patient's condition improved considerably with diminution of the follicular spines, regrowth of eyebrow hair and reduction of the thickened skin of the ears and nose (**Figure 1D**), supporting the original diagnosis.

### Isolation, cloning and sequencing of the viral genome

**Figure 2:**
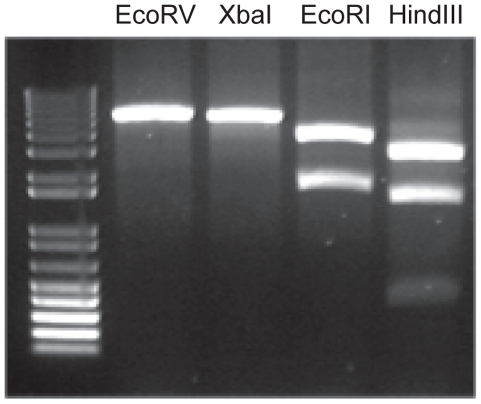
Restriction analysis of the RCA product. *Eco*RV and *Xba*I digestion revealed one band of around 5000 bp. *Eco*RI digestion produced two bands of around 3600 and 1600 bp. After *Hind*III digestion 3 bands were visible of around 3000, 1500 and 400 bp. Sequence analysis later showed that *Hind*III digestion in fact produced four fragments of which the smallest, 378 and 364 bp, coincided on gel.

Combined with what has been described in the literature, the above observations indicated that the patient could carry a polyomavirus causing TS. To identify this virus, spicules collected from the nose were dissolved in lysis buffer and subjected to nucleic acid extraction. The extracted material was used as a template for rolling-circle amplification (RCA) [15]. Instead of taq-polymerase used in conventional PCR, the RCA-method employs the DNA-dependent φ29-polymerase, a proofreading enzyme that preferentially amplifies circular DNA while using random primers. The RCA product was cut with restriction enzymes and analyzed on gel revealing a number of bands (**Figure 2**). Based on the size of these fragments, the amplicon was estimated about 5000 base pairs (bp) in length. This size is typical for polyomavirus genomes; papillomavirus commonly have larger genomes of around 8000 bp. The presence and location of the specific enzyme restriction sites was later confirmed when the viral sequence was elucidated.

The 3600 and 1600-bp RCA fragments, obtained after *Eco*RI digestion (Figure 2), were ligated and cloned into plasmid pUC19. Sequencing of each fragment was started from primers located up and downstream of the pUC19 multiple cloning site. Sequential sequence reactions were performed on the cloned RCA fragments, each time using newly designed primers based on the previously obtained sequence. A list of primers used for this “primer walking” is shown in Table S1. Finally, all obtained sequences were assembled into one continuous (circular) DNA contig of 5232 nucleotides. With the use of the newly designed primers, the resolved sequence was verified and confirmed in the original RCA product by direct sequencing.

### TSV genome analysis

**Figure 3:**
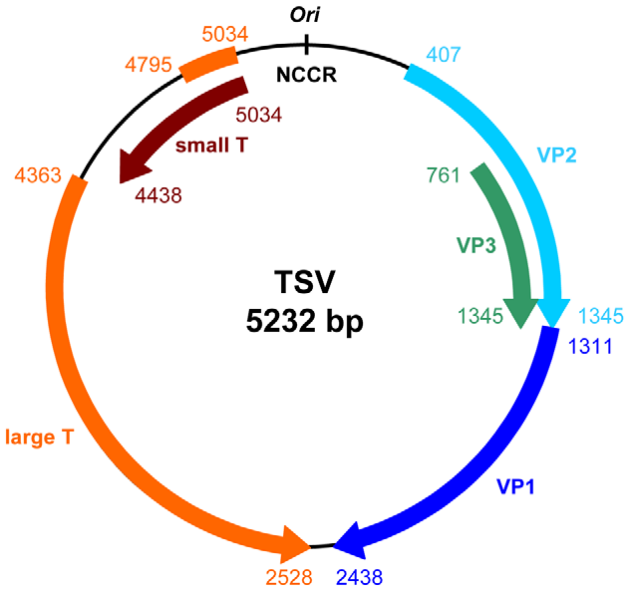
Genome map of TSV. Indicated are the five identified ORFs representing the putative “early” genes encoding small and large T antigen, and the putative “late” genes encoding VP1, VP2 and VP3. The NCCR is placed on top and contains the putative ori. Nucleotide position 1 was chosen within the NCCR in the large T binding region. For a detailed view of the NCCR, see Figure 4A.

Blast-mediated GenBank searches using the obtained 5232-bp circular DNA sequence as query consistently identified polyomaviruses as having most similar sequences. Analysis of this putative new viral genome revealed the presence of several open reading frames (ORFs) located on both strands. The orientation and relative size of these ORFs, as well as the presence of a non-coding control region (NCCR) in between, were similar to those of known polyomaviruses (**Figure 3**). Downstream of the NCCR that contains the origin of replication (ori), three putative late genes, VP1, VP2 and VP3, could be identified. Upstream of the NCCR, on the opposite strand, reside the candidate genes encoding the viral T antigens.

**Figure 4:**
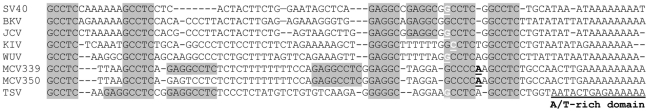
Detail of the TSV non-coding control region. Indicated are the putative large T-bindings sites located on both strands (gray-shaded boxes), the putative ori and putative TATA box (A/T-rich elements). Downstream of this area another two TSV putative large T-bindings sites are located, as well as one from KIV [1]. Nucleotide positions 1 are shown in white and underlined, except for MCV where the position 1 is shown in bold and underlined. For MCV two isolates are shown, MCV 339 and MCV350 [3].

In the NCCR, a total of ten putative large T-binding sites could be identified, seven and three respectively on each strand (**Figure 4**). An A/T-rich domain, probably harboring the TATA box, is located downstream of the last large T-binding site. By analogy with most other human polyomaviruses and SV40, nucleotide position 1 of the genome was chosen within the NCCR, in large T-binding region.

Because of obvious similarities in genome length, organization and sequence, we propose to group this newly identified TS-associated virus among the polyomaviruses. Nucleotide positions, length and estimated mass of all putative viral genes and proteins, respectively, are listed in **Table 1**. The level of protein sequence similarity with other known (human) polyomaviruses in shown in **Table 2**. The TSV genome and putative gene sequences, as well as the putative protein amino acid sequences have been submitted to GenBank (accession number GU989205).

**Table 1:** Overview of putative TSV genes and proteins.

TSV	Coding region (nt #)	GC content (%)	Amino acid number (n)	Calculated mass (kDa)
VP1	1311-2438	43,0	376	41,6
VP2	407-1345	43,0	313	34,9
VP3	761-1345	43,2	195	22,9
Small t	5034-4438	35,3	199	23,7
Large T 1	5034-4795, 4363-2528	37,0	692	79,7
Large T 2	5034-4795, 4381-2528	37,0	698	80,3

For each of the putative TSV genes the coding region and GC-content is shown. For the putative TSV proteins the amino acid number and the estimated mass are shown.

**Table 2:** Amino acid sequence similarities between putative TSV proteins and those of other polyomaviruses.

TSV protein	Amino acid sequence similarity (%)						
	JCV	BKV	KIV	WUV	MCV	SV40	OPyV1
VP1	50,7	52,5	21,9	24,1	50,6	52,9	77,7
VP2	33,0	33,0	13,9	13,0	30,0	32,0	88,3
VP3	32,8	32,2	12,4	11,4	22,9	31,1	88,7
Small t	34,0	33,2	32,0	30,4	34,7	33,7	70,9
Large T 1	42,6	41,1	44,0	43,9	42,0	39,6	87,7
Large T 2	42,3	40,8	43,6	43,7	43,0	39,3	88,4

For each of the putative TSV proteins, the amino acid sequence similarity is shown in comparison to the proteins of other known human polyomaviruses, SV40 and the Bornean orangutan polyomavirus (OPV1).

### TSV T antigen analysis

Like in all other polyomaviruses, the putative TSV small and large T antigens are expressed from a common primary transcript subject to alternative splicing [16], [17] (Figure 3), and share their N-terminal part of 80 amino acids in length. The most likely large T splice products were compared to the large T amino acid sequences of other known polyomaviruses producing two possible versions of the TSV large T antigen that differ only 6 amino acids in size. Version 1 was used for further analyses (Tables 1 and 2).

**Figure 5:**
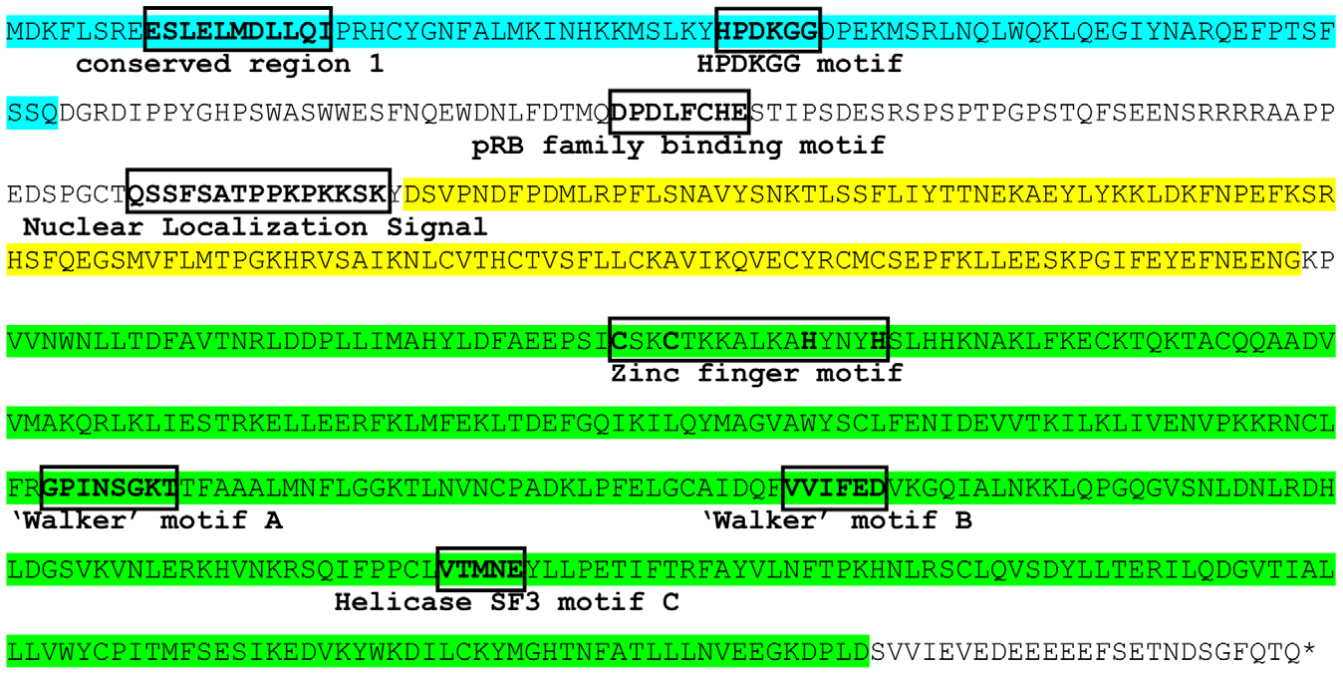
Amino acid sequence of the TSV large T protein. Indicated are three major domains found in the large T amino acid sequence; the J-domain (blue-shaded box), the ori DNA-binding domain (yellow-shaded box) and the helicase domain (green-shaded box). Within the J-domain the putative locations of conserved region 1 and the HPDKGG motif are depicted. Downstream of the J-domain, a pRb family-binding motif and nuclear localization signal are located. In the helicase domain, a zinc finger motif, NTPase-binding ‘Walker’ motifs A and B and an helicase SF3 (superfamily 3) motif C are located.

The putative TSV large T antigen contains characteristic sequence motifs in different domains, such as the J-domain, the ori DNA-binding domain and an ATPase/helicase domain (**Figure 5**). Within the N-terminal J-domain the highly conserved HPDKGG amino acid sequence is located, important for efficient polyomavirus DNA replication, transformation and virion assembly [18], [19]. Other characteristic polyomavirus large T sequence signatures are the pRb family-binding motif and a nuclear localization signal downstream of the J-domain [18]. In the ATPase/helicase domain, a zinc finger motif is recognized, two NTPase/helicase “Walker” motifs and an SF3 motif [20]. A sequence putatively involved in p53 complex formation that matches the p53-binding motif described by Pipas and coworkers (GPX_1_X_2_X_3_GKT [18]), overlaps with the “Walker” A motif located within the ATPase/helicase region shown in Figure 5 [19], [21]. In addition to the N-terminal J-domain shared with the large T antigen and similar to other known polyomaviruses, the putative TSV small T antigen contains protein phosphatase 2A subunit-binding motifs (not shown) [19], [21].

### Phylogenetic analysis

**Figure 6:**
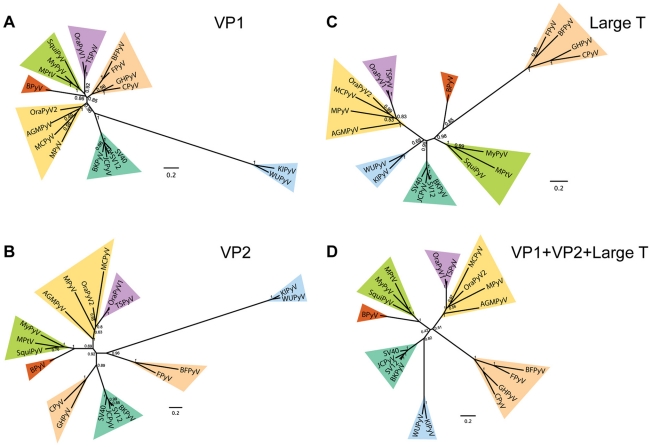
Phylogenetic analysis of known polyomaviruses and TSV. Bayesian posterior probability trees are shown for VP1 (A), VP2 (B), large T antigen (C) and a concatenation of all three (D). Numbers at branch points represent posterior probability support values and the scale bar is given in average number of substitutions per amino acid position. Major clades in the trees are highlighted using colored triangles. The following viruses are shown: Simian virus 40 (SV40), Goose hemorrhagic polyomavirus (GHPyV), Simian virus 12 (SV12), Squirrel monkey polyomavirus (SquiPyV), Finch polyomavirus (FPyV), Crow polyomavirus (CPyV), Bovine polyomavirus (BPyV), Merkel cell polyomavirus (MCPyV), WU Polyomavirus (WUPyV), KI polyomavirus Stockholm 60 (KIPyV), Budgerigar fledgling polyomavirus (BFPyV), African green monkey polyomavirus (AGMPyV), JC polyomavirus (JCPyV), BK polyomavirus (BKPyV), Murine polyomavirus (MPyV), Murine pneumotropic virus (MuPtV), Myotis polyomavirus VM-2008 (MyPyV), Bornean orangutan polyomavirus isolate Bo (OraPyV1), Sumatran orangutan polyomavirus isolate Pi (OraPyV2) and Trichodysplasia spinulosa-associated polyomavirus (TSPyV).

Complete sequences of 20 polyomavirus genomes were selected to represent the *Polyomaviridae* family in the RefSeq database [22]. They include 5 human, 4 avian and 11 non-human mammalian polyomavirus species. Except for the hamster polyomavirus (sequence incomplete) and Simian agent 12 (almost identical to simian virus 12), all of these plus the genome of the recently sequenced Sumatran orangutan polyomavirus [23] species were included in our analysis. We produced multiple sequence alignments and conducted phylogenetic analyses to determine the evolutionary position of TSV with respect to other members of the family (**Figure 6**).

The phylogenetic analysis involving VP1, VP2 and large T antigen, as well as a merged set of these proteins, recognized seven clades among polyomaviruses (colored triangles in Figure 6). In all trees analyzed, TSV was found to form a tight monophyletic cluster with the Bornean orangutan polyomavirus (OraPyV1) (violet clade in Figure 6). The distances separating these two closely related viruses resemble those found between JCV, BKV, SV40 and SV12 that form another compact monophyletic cluster (dark green clade in Figure 6). These findings support the classification of TSV as a new polyomavirus species rather than a strain of any of the known species.

In trees based on the VP2, large T antigen or the combination of VP1, VP2 and large T proteins, TSV and OraPyV1 are found within a monophyletic group formed by MCV, Sumatran orangutan polyomavirus, murine polyomavirus and African green monkey polyomavirus (yellow clade in Figure 6). The position of the TSV/OraPyV1 branch within this clade somewhat varies between trees, but consistently splits off the cluster trunk after the basal African green monkey polyomavirus lineage. In the VP1 tree, TSV and OraPyV1 form a separate clade, although this separation should be viewed with some caution since VP1 is the least conserved protein and the lineage branching in the yellow cluster is poorly resolved (Figure 6).

### TSV prevalence

The rare occurrence of TS suggests the (sub-clinical) circulation of TSV in larger populations outside this patient cohort. To investigate this possibility we developed three TSV-targeted quantitative PCR assays. Primers and probes were chosen in the VP1 and Large T genes, and in the NCCR, respectively, and listed in Table S2. As expected on the basis of chosen primer and probe sequences, none of the TSV PCRs recognized any of the other human polyomaviruses when performed on JCV-positive cerebrospinal fluids (n = 5), BKV-positive blood plasmas (n = 20), KIV or WUV-positive respiratory samples (n = 20) and MCV-positive plucked eyebrow hairs (n = 30)(data not shown). In contrast, each TSV PCR detected the presence of TSV DNA in the patient's biopsy fragment and in the plucked spicules from the nose. Calculation of the viral load in both samples revealed a mean TSV copy number of 2×10^5^ per cell.

To estimate the prevalence of TSV in immunosuppressed hosts, we analyzed a set of plucked eyebrows from long-term immunosuppressed renal transplant patients. Three out of 69 transplant patients (4%) were TSV-positive. The viral copy numbers detected in the plucked hairs of three of these patients were below one TSV copy per cell and, therefore, much lower than those detected in the patient's biopsy and spicules. Analysis of the patient's plucked eyebrow hairs collected six months after cidofovir treatment revealed a TSV load of 10^4^ copies per cell in all three PCRs. Spicules collected from untreated solitary lesions located on the legs contained amounts of TSV comparable to those detected in the spicules from the patient's nose before treatment, 3×10^5^ copies/cell.

## Discussion

With the discovery of TSV the total number of described human polyomaviruses is brought to six. Pathogenicity profiles have been established for JCV and BKV, to some extent for MCV, but not yet for KIV and WUV [24], [25]. Although not definitive, the evidence presented for TSV with regard to the clinical entity of TS can be considered reasonably strong. Previously, electron micrographs have shown the presence of a polyoma-like virus in nuclei of inner root sheath cells [8], [11], [13], [14]. These cells lay at the base of the distended and enlarged hair follicles that give rise to papule and spicule formation, the clinical hallmarks of the disease. From these spicules we could isolate the TSV genome and detect large amounts of the virus.

The rapid response of clinical signs to cidofovir treatment, also suggested a causal relationship between TSV and disease. Concomitantly a reduction in viral load was observed, but not as pronounced as expected based on the clinical response. To what extent TSV loads measured in plucked hairs and in spicules can be compared, and whether the difference in load measured between the two reflect the actual reduction in TSV load is unclear at the moment. Whether a threshold exists in TSV load, above which the clinical signs of TSV infection start to develop, is not known. Detection of TSV in high copy numbers in samples from earlier reported TS cases should provide further evidence for the pathogenicity of TSV.

Over the last years, different and often highly sophisticated methods for the detection of previously unknown viruses have been developed. RCA is a rather simple technique that takes advantage of the property of φ29-polymerase to preferentially amplify circular DNA while using random primers [15]. By strand displacement synthesis, a high molecular-weight DNA is produced containing multiple linear copies of the circular genome. The validity of this approach was demonstrated by the discovery of a number of newly identified papilloma and polyomaviruses [26], [27], [28], [29], [30]. Limitations of this method with respect to the minimal excess amount of viral over genomic DNA or maximum length of the viral genome are not exactly known. The absence of background smears or bands in our preparation shown in Figure 2 suggested a relative excess of circular (viral) DNA within the RCA product preparation, which was confirmed by qPCR on the spicules.

Analysis of the TSV genome revealed five putative genes probably encoding the VP1, VP2 and VP3 capsid antigens and the small and large T antigens. For the latter we have identified two putative splice variants, large T antigen 1 and 2. We found no ORF upstream of the TSV VP2 gene, indicating the absence of an LP1/Agno protein. Also the presence of alternative T proteins, such as middle T, seems unlikely. TSV lacks a third ORF within the T-antigen coding region as found in rodent polyomaviruses that encodes a middle T antigen, and no corresponding splice junctions were found. Further experimental investigation of TSV transcription is required to elucidate which (additional) TSV genes are expressed and how this is regulated.

Within the TSV large T protein several characteristic motifs could be located, including those required for binding the tumor-suppressor proteins pRb and p53. As shown for other well characterized polyomaviruses and also for papillomaviruses, binding and inactivation of these proteins promote cell transformation [19], [21], [31], a property that to some extent is shared by these small DNA (tumor) viruses. For high-risk human papillomaviruses oncogenicity has been established in the development of anogenital carcinomas. Integration of MCV probably plays a role in Merkel-cell carcinoma development [3]. If TSV possesses transformational properties and may play a part in carcinogenesis remains to be studied. In that respect, it is necessary to sort out whether TSV is potentially involved in other (hyper)proliferative (skin) diseases as well. For MCV for instance, observations have been made supporting a role also in development of cutaneous squamous-cell carcinomas [32].

Phylogenetic analysis of 20 fully sequenced polyomaviruses, including TSV, suggests the existence of seven polyomavirus clades. In all trees in Figure 6, substantial protein and virus-specific differences in branch lengths representing evolutionary distances were observed, e.g. between KI and WU polyomavirus in the VP1 and VP2 tree, and goose hemorrhagic, crow, finch and budgerigar fledgling polyomavirus in the large T antigen tree. In combination with some topological incongruence of the three protein-specific trees, these observations are indicative of a complex evolutionary history for most polyomaviruses.

OraPyV1 was isolated from blood of wild-caught and housed Bornean orangutans. Potentially, the properties of this virus, which remains poorly characterized beyond the genome sequence [23], could be insightful for understanding TSV. The murine and African green monkey polyomaviruses were isolated from leukemic extracts [33] and lymphoblastoid cells [34], respectively, also indicative of systemic infection. MCV was isolated from a Merkel-cell carcinoma and has been detected in other samples as well, including healthy skin biopsies, squamous-cell carcinomas, plucked hairs, and recently in respiratory samples as well [4], [32], [35], [36], [37], [38]. Initial attempts to detect TSV in other than skin-derived materials, such as blood plasma or serum, urine, cerebrospinal fluid failed. So far, we could detect TSV only in trichodysplasia tissue, spicules and plucked eyebrow hairs suggestive of a tropism specific for squamous epithelium, but larger studies are needed to confirm this finding.

Although the occurrence of TS is limited to severely immunocompromized patients, this implies that TSV would be present in larger, probably immunocompetent populations as well. By analogy with most human polyomaviruses, one could anticipate that TSV is highly immunogenic and infects many people, probably early in life without apparent disease [39], [40]. Further (sero)epidemiological studies have to reveal if this indeed is the case and whether TSV causes low-level persistent (latent) infection, as described for other polyomaviruses.

Although we have tested thus far only a limited number of individuals, TSV was detected in another three unrelated immunosuppressed patients without signs of trichodysplasia. This would be compatible with occasional infections from an unknown reservoir. In view of the lack of any epidemiological marker of the disease, however, it seems more likely the virus is common among humans but generally without causing disease, comparable to for instance JCV and BKV. In that case, at least in adults and in plucked eyebrow hairs, (latent) TSV loads may often be too low to be detected by our current assays. Although the eyebrow region is particularly affected in TS and eyebrow hairs were shown suitable material to detect (polyoma)viruses [4], [41], with 50% MCV-positivity in this study (data not shown) comparable to what Wieland and coworkers have found [4], it is not known at the moment whether eyebrow hairs represent a suitable clinical sample to detect low level TSV infections.

In conclusion, a new human polyomavirus was discovered and identified as the possible cause of TS in an immunocompromized patient. We provided evidence for the presence of TSV also among unaffected patients suggestive of subclinical, possibly latent infection. Additional studies in different populations and age groups using different clinical materials are needed to establish the (sero)prevalence and epidemiology of TSV infections, and its possible relation to the occurrence of other (skin) diseases, including cancer. For a general picture, these epidemiological studies should be complemented with experimental studies on TSV replication, transcription and transformation.

## Materials and Methods

### Ethics statement

The TS patient and his mother gave oral consent to collect spicules and eyebrow hairs for viral diagnosis and treatment monitoring. The Medical Ethics Committee of the LUMC declared in writing that no formal ethical approval was needed to analyze these clinically obtained materials. Written consent from the patient (minor) and his legal guardian (mother) was obtained for publication of his case and for showing his pictures.

Plucked eyebrow hair samples from a population of renal transplant patients visiting the Dermatology outpatient clinic of the LUMC were obtained after informed oral consent from the subjects, which was documented in the patient files. Subject's written approval was not collected for this purpose (plucking of eyebrow hairs), as oral consent was considered appropriate in this case by the Medical Ethics Committee of the LUMC who approved of the study (Protocol P07.024: Risk factors for non-melanoma skin cancer/Genetic and environmental risk factors for the development of skin cancer in organ-transplant recipients).

### Sample collection and DNA isolation

Ten spicules from the nose collected with sterile tweezers in a sterile vial were shipped to the Leiden University Medical Center (LUMC) at room temperature. Upon arrival the plugs were dissolved in a proteinase K-containing lysis buffer for overnight incubation at 56°C. Total DNA was isolated with the QIAamp DNA Mini Kit (Qiagen) according to the QIAmp tissue protocol, with some minor alterations [42]. In parallel, approximately 100 ng commercially available human genomic DNA (Promega) was extracted as a negative isolation control.

### Rolling circle amplification (RCA)

The TempliPhi 100 RCA Kit (GE Healthcare, UK Limited) was used following manufacturer's instructions with some slight modifications. In brief, 1 µl, 1∶100 of the isolated total DNA, was diluted in 5 µl sample buffer, denatured at 95°C for 3 minutes and cooled down slowly to 4°C to allow primer annealing. Meanwhile a premix of 5 µl reaction buffer, 0.2 µl TempliPhi enzyme (bacteriophage ϕ29 DNA polymerase) and an extra 450 µM of each dNTP was prepared and added to the denatured DNA in sample buffer. The RCA reaction was preformed at 30°C for 16 hours followed by inactivation of the enzyme at 65°C for 10 minutes. The RCA product was stored at −20°C.

### Genomic sequencing, assembly and analysis

The RCA product was diluted 1∶1 in miliQ H_2_O and 2 µl was digested with *Eco*RV, *Hind*III, *Eco*RI or *Xba*I. The two fragments of the *Eco*RI digestion were isolated from gel, ligated and cloned into pUC19, and subsequently sequenced using M13 forward and reverse primers. The resulting sequences were used as a template to design new primers located at the end of the newly identified sequences and listed in Table S1. Sequence reactions were carried out with the BigDye Terminator kit (Applied Biosystems) and analyzed on an ABI Prism 3130 Genetic Analyzer (Applied Biosystems).

Contig sequence assembly was performed with ContigExpress, included in the vector NTI software package program, that uses CAP3 computations to drive the assembly process [43]. Putative splice donor and acceptor sites were identified based on consensus splice donor and acceptor sequences as published [44], [45], and automated splice-site predictions (http://zeus2.itb.cnr.it/~webgene/wwwspliceview.html). Putative large T binding sites within the NCCR were identified according to described motifs [46].

Domain searches within the TSV large and small T antigen sequences were performed against the domain profile database SCOP [47] using the HHsearch software [48]. Hits against all 3 domains were strongly significant (E-values <E-12).

### Sequence alignments and phylogenetic analysis

Amino acid sequence similarities between the putative TSV gene products and those of other polyomaviruses, as shown in Table 2, were calculated with the AlignX program in vector NTI version 11, which uses the ClustalW algorithm with default alignment parameters.

For the phylogenic analyses all available polyomavirus genome sequences present in the RefSeq database in December 2009 were downloaded [22]. The Sumatran orangutan polyomavirus [23] and the identified TSV genome sequences were added to this set. The following genome sequences were included in the analysis: Simian virus 40 (NC_001669), Goose hemorrhagic polyomavirus (NC_004800), Simian virus 12 (NC_012122), Squirrel monkey polyomavirus (NC_009951), Finch polyomavirus (NC_007923), Crow polyomavirus (NC_007922), Bovine polyomavirus (NC_001442), Merkel cell polyomavirus (NC_010277), WU Polyomavirus (NC_009539), KI polyomavirus Stockholm 60 (NC_009238), Budgerigar fledgling polyomavirus (NC_004764), African green monkey polyomavirus (NC_004763), JC polyomavirus (NC_001699), BK polyomavirus (NC_001538), Murine polyomavirus (NC_001515), Murine pneumotropic virus (NC_001505), Myotis polyomavirus VM-2008 (NC_011310), Bornean orangutan polyomavirus isolate Bo (FN356900), Sumatran orangutan polyomavirus isolate Pi (FN356901) and Trichodysplasia spinulosa-associated polyomavirus (GU989205).

Multiple amino acid alignments were compiled for VP1, VP2 and large T antigen using the Muscle program [49], followed by manual inspection assisted by the Viralis software platform [50]. For VP2 and large T antigen, only partial alignments were used covering, respectively, the part not overlapping with VP1 (positions 407 to 1307 in the TSV genome sequence) and the large exon including the helicase domain (positions 4130 to 2600 in the TSV genome sequence). The three protein-specific alignments and their concatenation were submitted to phylogenetic analyses.

Bayesian posterior probability trees were compiled utilizing the BEAST software [51]. MCMC chains (two per dataset) were run for 2 million steps (10% burn-in, sampled every 50 generations) under the WAG amino acid substitution model [52], and rate heterogeneity among sites (gamma distribution with 4 categories). For each analysis three molecular clock models (strict, relaxed with lognormal distribution, relaxed with exponential distribution) were tested [53]. The more complex model, e.g. relaxed molecular clock, was favored over the simpler model, e.g. strict molecular clock, if the Bayes factor (ratio of tree likelihoods) was bigger than five [54]. Convergence of runs was verified and Bayes factors were estimated using Tracer software (http://beast.bio.ed.ac.uk/Tracer).

### PCR development and testing

For the detection of TSV DNA, three real-time quantitative PCRs were developed with primers and Taqman probes located in the NCCR, and the VP1 and Large T ORFs, respectively (Table S2). Primers and probes were chosen with the help of Beacon Designer software (Premier Biosoft). The VP1 3′ primer had a 84% match with BKV, but none of the chosen TSV probes had similarities with any of the other known polyomaviruses.

The 50 µl PCR reactions consisted of 1× GeneAmp PCR buffer (15 mM Tris-HCl [pH 8,0], 50 mM KCl, 3,6 mM MgCl, 0,3 mM of each dNTP, 15 pmol of each primer, 7,5 pmol probe and 2 U of AmpliTaq Gold polymerase (Applied Biosystems). Real-time PCR was performed in the iCycler (Biorad) and cycle conditions are 9′ at 95°C, followed by 50 cycles of amplification (94°C for 1 min. and 65°C for 1 min.). TSV copy number was calculated against a plasmid titration series of pUC19-TSV included in each PCR assay that contains the full length TSV genome cloned in *Xba*I. Cell number was calculated with a PCR specific for the beta-actin gene, which was run in parallel on a dilution series of human genomic DNA (Promega). The sensitivity of each TSV PCR was found to be between 1–10 TSV genome copies.

The cerebrospinal fluids, blood plasmas and respiratory samples with proven JCV-, BKV-, KIV- or WUV-positivity used for validation of the developed TSV-specific PCRs were selected from clinical samples routinely send for viral diagnosis to the LUMC, Dept of Medical Microbiology. The plucked eyebrow hair samples were obtained with written permission from renal transplant patients visiting the Dermatology outpatient clinic of the LUMC.

## Supporting Information

Table S1 List of TSV primers used for primer-walking and sequencing.
(0.49 MB TIF)

Table S2 List of TSV primers and probes used for quantitative PCR.
(0.24 MB TIF)
