# Supplementary figures and images for: Discovery of a New Human Polyomavirus Associated with Trichodysplasia Spinulosa in an Immunocompromized Patient
Source: PLoS Pathog. 2010 Jul 29;6(7):e1001024. doi: 10.1371/journal.ppat.1001024 (PMC2912394; doi:10.1371/journal.ppat.1001024)

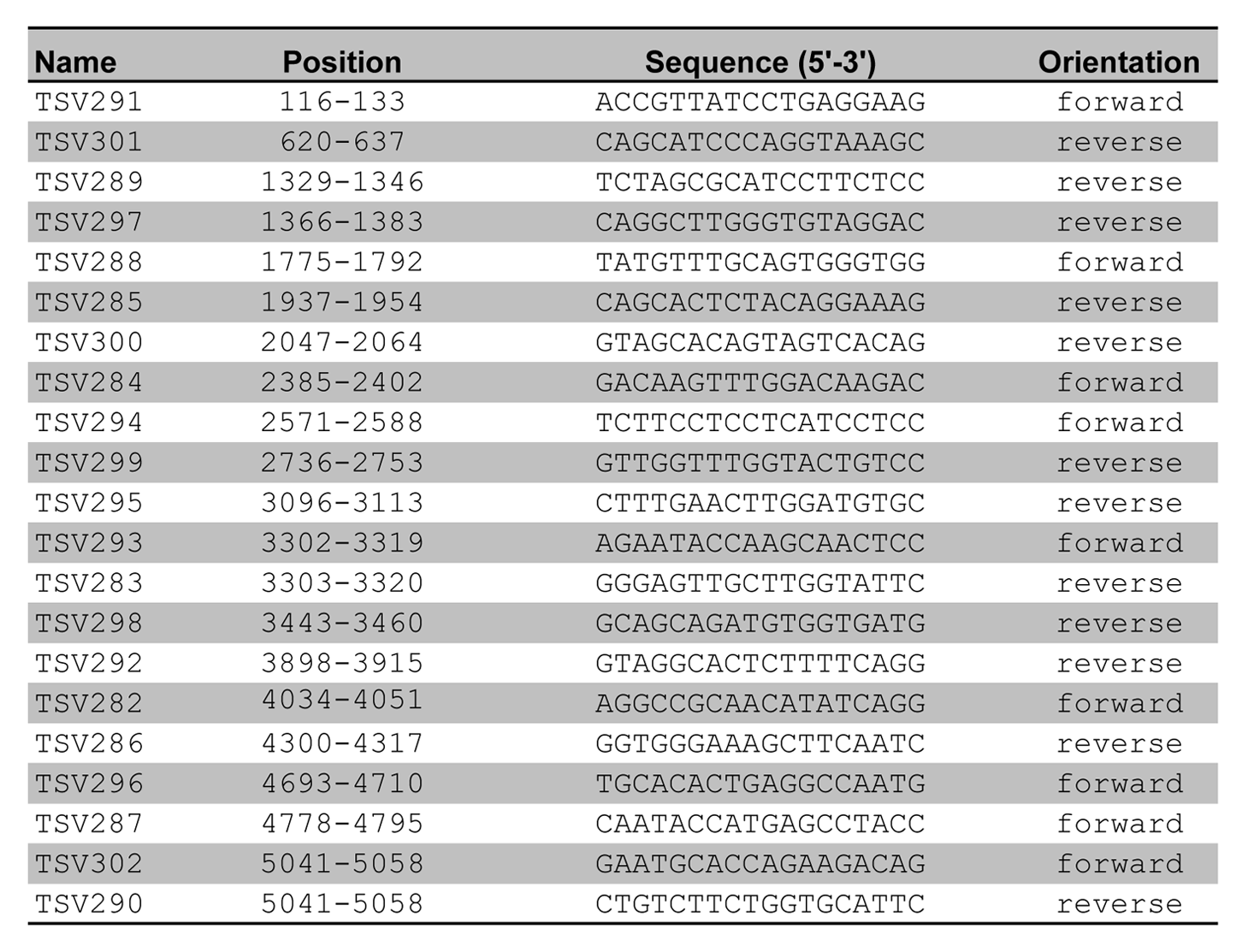

Supplement: Table S1 — List of TSV primers used for primer-walking and sequencing. (0.49 MB TIF) [file ppat.1001024.s001.tif]

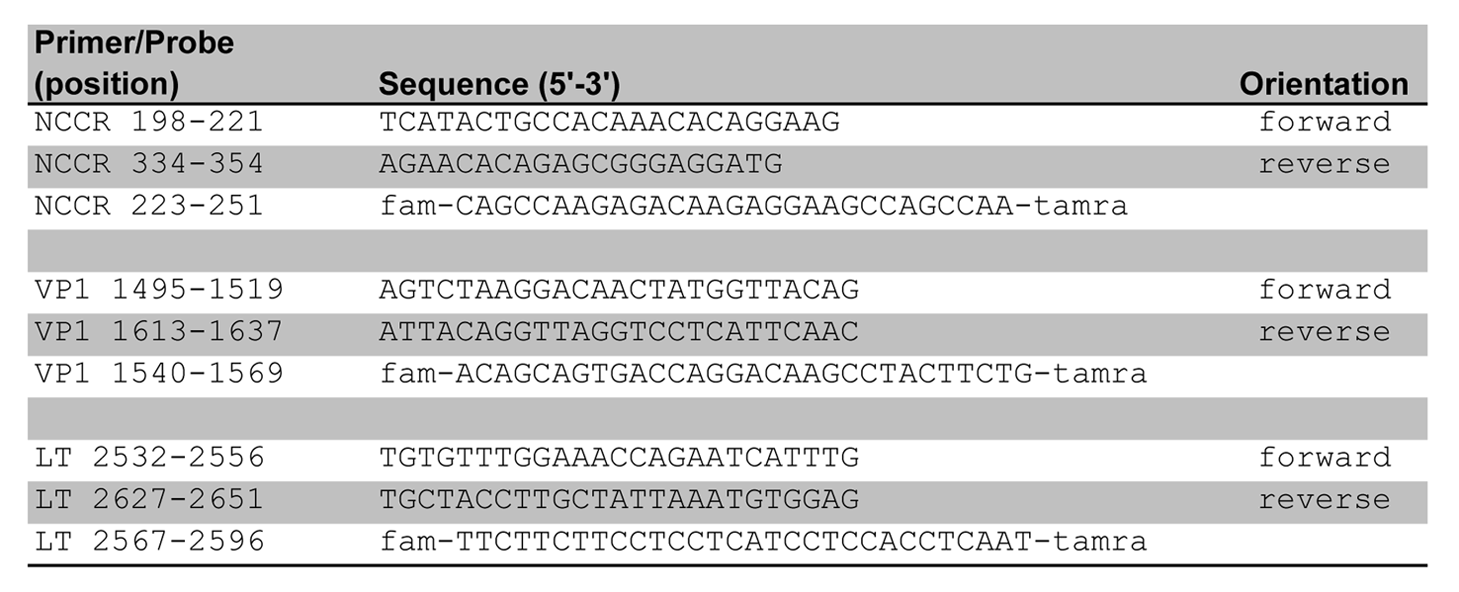

Supplement: Table S2 — List of TSV primers and probes used for quantitative PCR. (0.24 MB TIF) [file ppat.1001024.s002.tif]
